# The mRNA of TCTP functions as a sponge to maintain homeostasis of TCTP protein levels in hepatocellular carcinoma

**DOI:** 10.1038/s41419-020-03149-7

**Published:** 2020-11-12

**Authors:** Wei Liu, Qi Liu, Beilei Zhang, Zhibin Lin, Xia Li, Xisheng Yang, Meng Pu, Rongzhi Zou, Zhiying He, Fu Wang, Kefeng Dou

**Affiliations:** 1 Institute for Regenerative Medicine, Shanghai East Hospital, Tongji University, Shanghai, 200123 China; 2 Department of Hepatobiliary Surgery, Xijing Hospital, Air Force Medical University, Xi’an, Shaanxi Province 710032 China; 3 Shanghai Institute of Stem Cell Research and Clinical Translation, Shanghai, 200120 China; 4 Department of Gynecology and Obstetrics, Tangdu Hospital, Air Force Medical University, Xi’an, Shaanxi 710038 China; 5 Institute of Biophysics, Chinese Academy of Science, Beijing, 100101 China; 6 Engineering Research Center of Molecular and Neuro Imaging, Ministry of Education, School of Life Science and Technology, Xidian University, Xi’an, Shaanxi 710071 China

**Keywords:** Cancer, Molecular biology

## Abstract

Translationally controlled tumor protein (TCTP) is a highly conserved protein that accumulated in the tumorigenesis of various malignancies. Despite the important role of TCTP protein in tumor progression, the precise function and underlying mechanistic regulation of TCTP mRNA in hepatocellular carcinoma (HCC) remain unclear. In this study, we found that TCTP protein was overexpressed in HCC patients but TCTP mRNA expression levels were reversed. TCTP knockout HCC cells exhibited attenuated abilities of proliferation, migration, and invasion. The knockdown of TCTP by siRNA effectively reduced TCTP mRNA levels but not protein levels in HCC cells. Moreover, although the constitutive knockdown of TCTP inhibited almost 80% of TCTP protein expression levels in tumors of wildtype transgenic mice (TCTP KD/WT), partial restoration of TCTP protein expression was observed in the tumors of heterozygous TCTP mice (TCTP KD/TCTP±). The blockage of mRNA synthesis with ActD stimulated TCTP protein expression in HCC cells. In contrast, combined treatment with ActD and CHX or MG132 treatment alone did not lead to the TCTP protein accumulation in cells. Furthermore, following the introduction of exogenous TCTP in cells and orthotopic HCC tumor models, the endogenous TCTP protein did not change with the recombinational TCTP expression and kept a rather stable level. Dual-luciferase assays revealed that the coding sequence of TCTP mRNA functions as a sponge to regulate the TCTP protein expression. Collectively, our results indicated that the TCTP mRNA and protein formed a closed regulatory circuit and works as a buffering system to keep the homeostasis of TCTP protein levels in HCC.

## Introduction

Hepatocellular carcinoma (HCC) is one of the most common malignancies and the leading cause of death in the world for years^1^. The chronic hepatitis B and C infection and prolonged exposure to hepatocarcinogens have been considered to be the ultimate cause of HCC^2^. Other risk factors, including inherited metabolic diseases, nonalcoholic steatohepatitis, and tobacco smoking, have also been proposed to induce HCC^3^. Due to the high recurrence rates of 70–80% within 5 years and low long-term survival rate of 7% within 10 years after resection^4^, the identification of specific targets for early diagnosis and therapy of HCC is imperative and urgent. Therefore, factors that can induce HCC reversion, which means expelling HCC cells to quit the malignancy, have been highly desired and explored for HCC therapy in clinic^5,6^.

Translationally controlled tumor protein (TCTP), a highly conserved protein, has been implicated as one of the most important biomarkers and targets for tumor reversion^7,8^. TCTP was downregulated at both mRNA and protein levels during tumor reversion^9,10^. By inhibiting the expression of TCTP, the numbers of revertant cells were raised to 30% in colon, lung, and melanoma cancers in vitro^11^. The increased accumulation of TCTP was also observed in the tumorigenesis of different types of cancers and correlated with poor prognosis^12^. In HCC development, the high expression level of TCTP was significantly associated with the advanced tumor stage (*P* = 0.037) and overall survival time of HCC patients (*P* = 0.034)^13^, indicating the critical role of TCTP in tumor progression and regression. Therefore, TCTP may represent a promising target for HCC prevention and intervention.

Multiple factors have been found to regulate the expression of TCTP at both transcription and translation levels, and the regulatory effects from the same factor may be inconsistent in different conditions^14,15^. The complexity and exquisite of the regulatory network may add uncertainty in targeting TCTP for cancer therapy. Moreover, the sequence of TCTP mRNA itself is highly structured and functional, displaying features typical of translationally controlled mRNAs^16^. The 5′-terminal oligopyrimidine tract (5′-TOP) in its 5′ untranslated region (5′-UTR) functions to repress its translation under quiescent conditions^17^. 5′- and 3′-UTR of TCTP mRNA has also been reported as targets for multiple chemical substances with different structures by transcriptional and post-transcriptional mechanisms^18^. Another interesting observation is that TCTP mRNA is capable of folding into a functional secondary structure with extended base-paired areas. Due to this secondary structure, TCTP mRNA is negatively regulated at the translational level through the double-stranded RNA-activated protein kinase PKR^19^. As well, in erythroleukemia and several other cell lines, TCTP mRNA has been found to accumulate to high levels without the accumulation of its corresponding encoded protein^20^. These findings implied that TCTP mRNA itself may be subjected to translational regulation and have a function other than just translating TCTP protein. However, the precise function and the underlying mechanistic regulation of TCTP are far from clear.

In the present study, we explored the regulatory mechanisms of TCTP mRNA involved in HCC progression. Our results indicated the existence of reciprocal regulation between TCTP mRNA and protein, which formed a closed regulatory circuit and kept TCTP protein at a rather stable level. The TCTP mRNA alone functions as a sponge: lower TCTP mRNA levels stimulated higher translation efficiency while higher mRNA levels repressed its translation. Our findings deciphered the unusual self-regulatory mechanisms for the known proto-oncogene TCTP, as well as the coding-independent regulatory function of TCTP mRNA.

## Material and methods

### Western blot and analysis

All cell lines were harvested and lysed in RIPA lysis buffer (Beyotime Biotechnology) at room temperature for 10 min. Related tissues were lysed in RIPA buffer and ground on ice for 30 s. The supernatants were collected and protein concentrations were determined using a BCA protein assay kit (Beyotime Biotechnology), 40 µg of protein were loaded per lane. Western Blots were performed following standard techniques. All antibodies were purchased from Cell Signaling technology. The immunoreactivity was detected using the ECL detecting instrument (GE Healthcare Bio-Sciences). Gel images were captured by Bioradchemidoc MP (BioRad) and the protein level was quantified by the associated Image Lab analysis software. Actin and Gadph were used as an internal reference.

### Patients and clinical specimens

Pairs of HCC tissues and adjacent normal tissues (*para*-tissue) were collected immediately after hepatectomy at the Forth Military Medical University Xijing Hospital (Xi’an, China) from 2010 to 2016. None of these patients received preoperative chemotherapy or radiotherapy. All HCC patients signed written informed consent on the use of clinical specimens for medical research. Samples used in this study were approved by the Committees for Ethical Review of Research at the Forth Military Medical University.

### Cell culture and transfection

HCC cell lines Huh7, HepG2, immortalized normal human liver cell line HL-7702, mouse HCC cell line Hepa 1–6, and lung cancer cell line A549 was obtained from the Chinese Academy of Medical Sciences (Beijing, China). Authentication of cells was verified by short tandem repeat DNA profiling. All cells were cultured in DMEM medium supplemented with 10% fetal bovine serum at 37 °C in 5% CO_2_ containing a humidified atmosphere. Plasmids or siRNAs were transfected with lipofectamine 2000 according to the standard protocol.

### Dual-Luciferase reporter assay

Luciferase reporter constructs (psiCheck2-CDS, psiCheck2-FL, psiCheck2-blank) were transfected into different cells using Lipofectamine 2000 (Invitrogen, Carlsbad, CA) according to the manufacturer’s instructions as described previously^21^. A Dual-luciferase kit (Promega, Madison, WI) was used to measure luciferase activities according to the manufacturer’s instructions with some modification. The reads of renilla luciferase were normalized to secondary luciferase-firefly luciferase, which was designed to be an internal control plasmid for normalization of transfection efficiency.

### Wound healing assay

Cells were seeded on 6-well plates and cell layers were scratched with a 200-μL pipette tip. After washing cells with phosphate-buffered saline to remove debris, the images of wound closure were captured at 0 and 24 h time points. All experiments were performed in triplicate.

### Cell migration and invasion assay

Cell migration was measured using the Transwell plate (Corning, NY, USA) with a 0.4-μm pore polyester membrane. Briefly, cells were suspended at a density of 1.0 × 10^6^/ml and 200 μl of cell suspension was transferred to the upper chamber. The bottom chamber was filled with 600 μl of complete medium. After 24 h of incubation, cells were immobilized with 100% methanol for 10 min and stained with 4 g/L crystal violet solution for 15 min. For cell invasion assay, the experimental procedures were similar to cell migration assay but an invasion chamber 24-well plate (Corning, NY, USA) was used instead of the Transwell plate. For both assays, migrated cells were counted in five randomly selected areas under a 100× microscope field. All assays were performed in triplicate.

### Xenograft tumor model

All animal experiments were performed with the Guild for the Care and Use of Laboratory Animals according to animal protocols approved by The Forth Military Medical University. Approximately, 5 × 10^6^ of TCTP-overexpressed Hepa1–6 cells, TCTP Cas9 KO Hepa1–6 cells, or siKD Hepa1–6 cells were suspended in 50 μl of serum-free DMEM and injected subcutaneously into the right or left dorsal flank of 4-week-old Balb/c female nude mice, respectively. The animals were randomized. Two weeks after injection, tumor volume was measured and calculated by the following equation: *V* = (*L* × *W*^2^)/2 (V, volume; L, length; W, width).

### Orthotopic tumor model and in vivo bioluminescence imaging

Approximately, 1 × 10^7^ of wild-type (WT) Hepa1–6 cells, TCTP-overexpressed Hepa1–6 cells, TCTP Cas9 KO Hepa1–6 cells, or siKD Hepa1–6 cells were suspended in 25 μl of serum-free DMEM and mixed with Matrigel, respectively. The mixture was then transplanted to the liver of 8-week-old C57BL female mice with different genotypes to establish an orthotopic mouse model. The animals were randomized into three groups. Optical in vivo imaging was taken two weeks after transplantation. d-Luciferin (Genomeditech) was intraperitoneally injected into mice at the concentration of 150 mg/kg 10 min before imaging. The mice were then anesthetized with 2% isoflurane and the bioluminescence imaging was taken using a Xenogen Lumina Π system for 5 min. The same exposure time was used in all mice. The luminescence intensity was analyzed using Living Image software 4.1 (Xenogen).

### Statistical analysis

All data were shown as median with standard deviation. The Mann–Whitney *U* test was used to compare continuous variables, and the Pearson chi-square test was used to compare discrete variables. SPSS Statistics version 19 was performed to calculate all statistical data. The independent Student’s *t* test was calculated to compare tumor size and luciferase activity between any two preselected groups. *P* < 0.05 was considered statistically significant.

## Results

### TCTP protein is essential for HCC progression

**Fig. 1:**
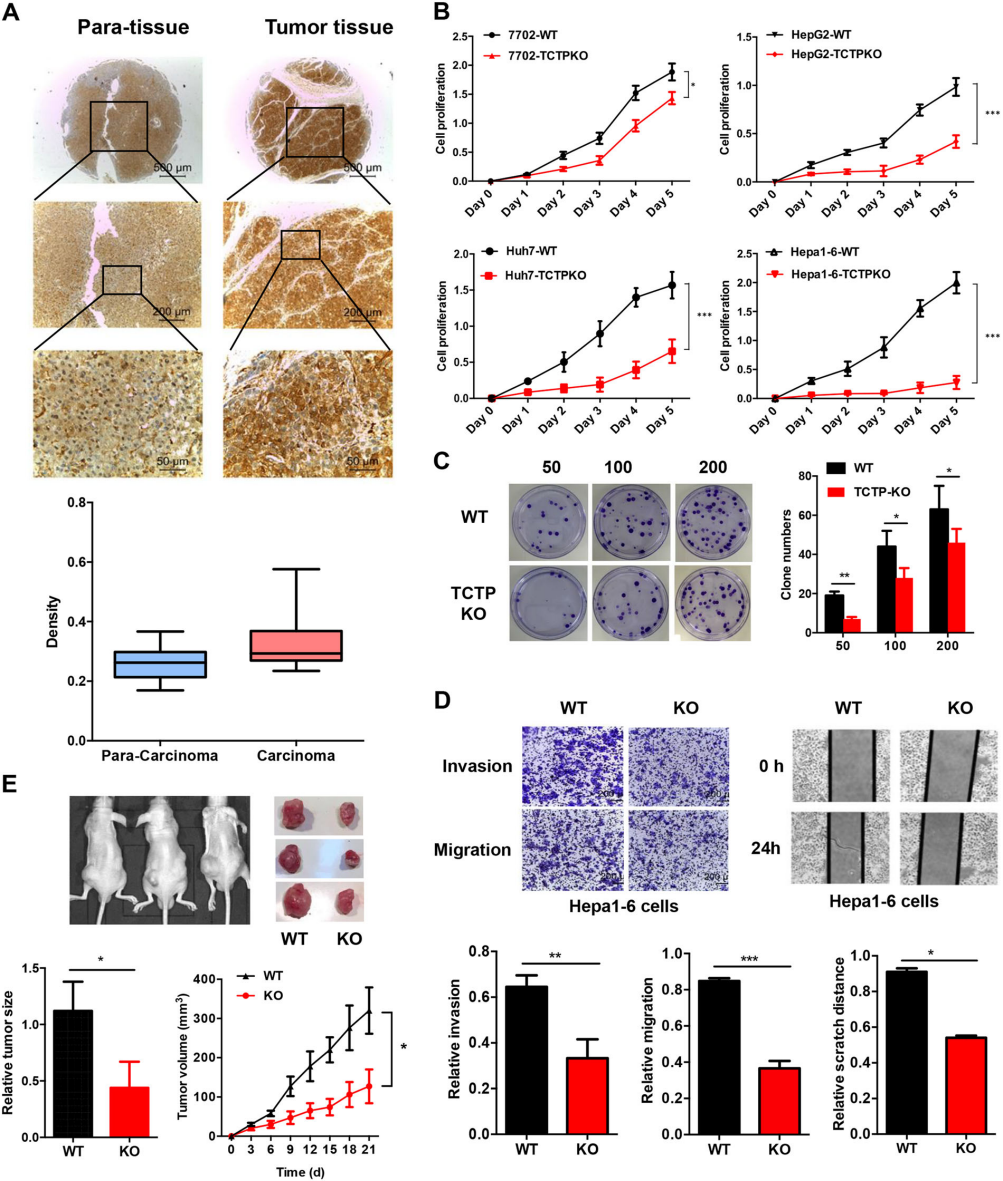
TCTP is essential for HCC progression in vitro and in vivo. **A** Representative images of IHC analysis of TCTP expression in paired HCC tumors and *para*-tissues. **B** CCK8 assay was used to determine the cell proliferation of wildtype and TCTP KO cells. **C** Different numbers of wildtype (WT) and TCTP KO Hepa1–6 cells were seeded in 6-well plates. Two weeks later, the numbers of survival colonies were calculated. **D** The migration and the invasion abilities of WT and TCTP KO Hepa1–6 cells were investigated by the Transwell invasion assay, Transwell migration assay, and wound healing assay. **E** WT and TCTP KO Hepa1–6 cells were planted into the left and right flanks of nude mice to establish a xenograft tumor model. Representative images of mice bearing tumors and tumor tissues from both flanks of mice (top). (Bottom) Quantification of tumor size and tumor growth curve between WT and TCTP KO cells implanted groups. The data are expressed as means ± sd. Statistical analyses using Student’s *t* test for independent groups were performed using GraphPad Prism5. *P < 0.05, **P < 0.01, ***P < 0.001.

According to the clinicopathological features of 49 HCC patients, involving age, gender, tumor size, and pathological grade, analysis of TCTP expression showed that TCTP expression was positively correlated to high pathological grades (*P* = 0.019) and tumor node metastasis stage (*P* = 0.006) (Table 1). High TCTP expression levels (defined as a greater than 50% increase compared with corresponding *para*-carcinoma tissue, normalized with GAPDH) were detected in 75.5% of HCC patients (37 of 49 of HCC cases). Immunohistochemical (IHC) staining of TCTP was conducted in samples from 13 HCC patients with different tumor stages (3 stage I, 5 stage II, 5 stage III). As shown in Fig. 1A, 70% of advanced HCC cases (stage II and III) showed an obviously higher expression of TCTP compared with *para*-carcinoma tissues (*P* < 0.01). We also found that the increase of phosphorylation of ERK and AKT was associated with TCTP overexpression in HCC tumors (Supplementary Fig. S1A), confirming that TCTP expression is regulated through PI3-K/Akt/mTORC1 signaling pathway^22^.

**Table 1:** Relationship between TCTP expression and clinical features of HCC patients.

Clinical features	Number	TCTP overexpression (%)	*P* value
Gender			0.721
Female	35	27 (77.2)	
Male	14	10 (71.4)	
Age (years)			1.254
≤60	35	26 (74.3)	
>60	14	11 (75.6)	
HBsAg			1.548
Positive	38	27 (71.1)	
Negative	11	8 (72.7)	
Serum AFP (ng/ml)			0.754
≤400	19	13 (68.4)	
>400	30	22 (73.3)	
Tumor size (cm)			0.183
≤5	23	15 (65.2)	
>5	26	22 (84.6)	
Pathological grades			0.019
Ι	4	2 (50.0)	
II	33	22 (66.7)	
III	12	11 (91.7)	
TNM stage			0.006
Ι–III	38	28 (73.7)	
IV	11	7 (63.6)	
Tumor stage (AJCC)			0.49
Stage Ι	7	5 (71.4)	
Stage II	32	23 (71.9)	
Stage III	10	9 (90.0)

To prove the essentiality of TCTP expression for HCC, we knockout (KO) TCTP in four hepatocyte lineages with CRISPR/Cas9 lentivirus system to establish four TCTP KO stable cell lines (Supplementary Fig. S1B). Lack of TCTP protein was strikingly linked to several cancer-related features in different hepatic cell lines, including human HCC cell lines (Huh7 and HepG2), human normal liver cell line (HL-7702), and mouse HCC cell line (Hepa1–6). Specifically, both the cell proliferation (Fig. 1B) and the colony formation abilities (Fig. 1C) were repressed in TCTP KO HCC cells. Moreover, cell migration and invasion were also inhibited in the TCTP-depleted Hepa1–6 cells (Fig. 1D) and HL-7702 cells (Supplementary Fig. S1C). And the repression effect on the proliferation of HCC cell lines was more severe than that on human normal liver cell line HL-7702.

To further confirm the role of TCTP in HCC tumorigenesis, the WT, and TCTP KO Hepa1–6 cells were subcutaneously planted into the nude mice to establish a xenograft tumor model. Compared with the WT Hepa1–6 cells, the TCTP KO cells almost lost the tumorigenicity in vivo with slower tumor growth and smaller tumors (Fig. 1E). TCTP and Ki-67 expression was detected in the WT and TCTP KO tumor samples by IHC. The results showed that Ki-67 expression was obviously decreased in the TCTP KO samples (Supplementary Fig. S1D), further indicating KO of TCTP leads to tumor growth retardation in vivo. Together, these results proved that TCTP protein is essential for HCC progression in vitro and in vivo.

### TCTP mRNA expression levels are opposite to its protein levels in HCC

**Fig. 2:**
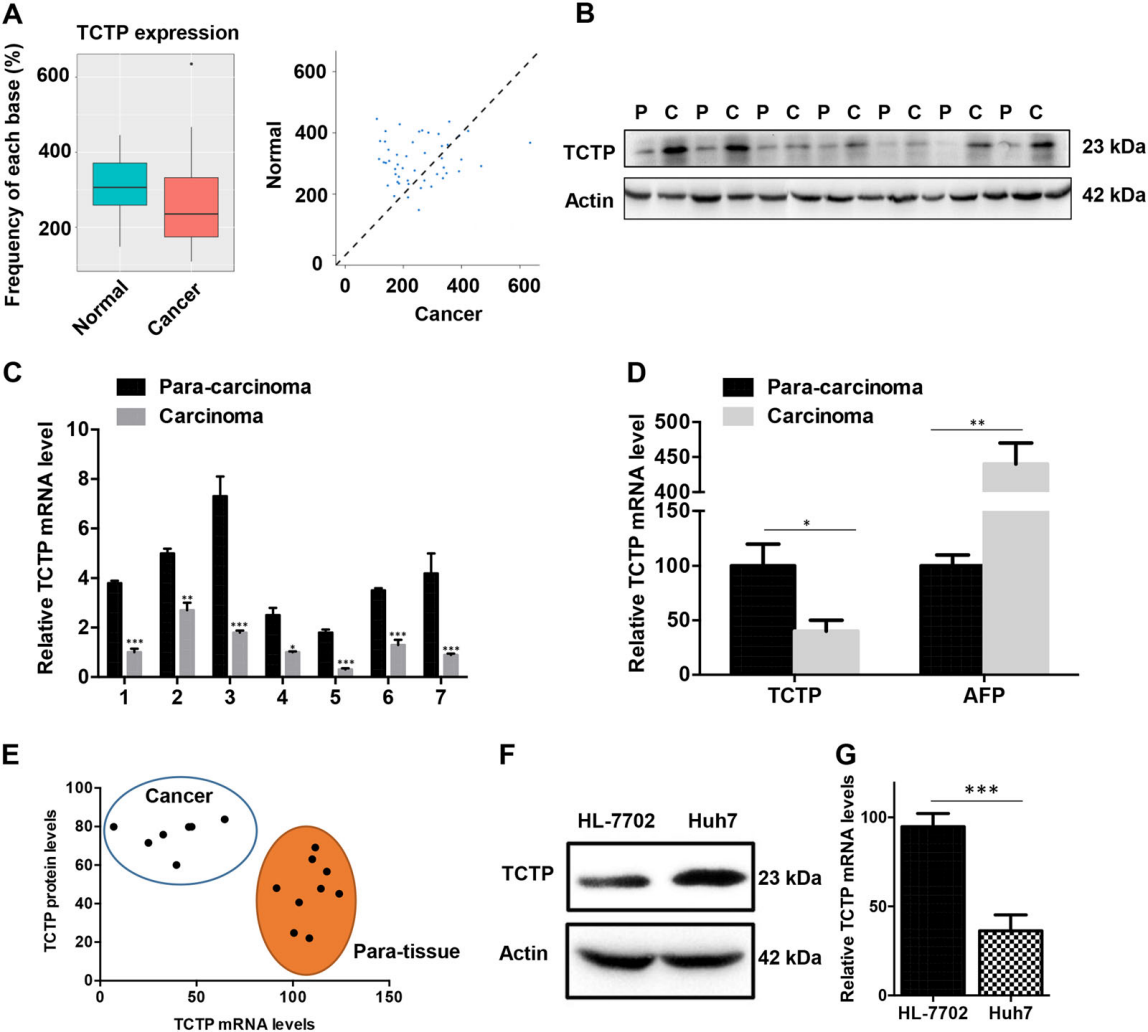
The contrary expression between TCTP mRNA and protein in HCC. **A** Bioinformatics analysis of samples in TCGA indicates a reverse trend of distribution between TCTP mRNA and its protein. The mRNA distribution difference between HCC and *para*-tissues is significant (*P* = 0.023). **B**, **C** Western blot (**B**), and qPCR (**C**) were used to examine the TCTP protein and mRNA expression in the HCC (indicated as C) and *para*-tissues (indicated as P). **D** The negative correlation between mRNA levels of TCTP and AFP in the HCC and *para*-tissues. **E** Quantification of TCTP mRNA level and protein levels in HCC tumors and *para*-tissues. **F**, **G** Western blot (**F**), and qPCR (**G**) were used to measure the TCTP protein and mRNA expression in HL-7702 and Huh7 cells. The experiments were performed in triplicate. The data are expressed as means ± sd. Statistical analyses using Student’s *t* test for independent groups were performed using GraphPad Prism5. *P < 0.05, **P < 0.01, ***P < 0.001.

After determining the high expression level of TCTP protein in HCC, we then asked whether TCTP mRNA is also upregulated in HCC. We utilized the cancer genome atlas (TCGA) online database (https://cancergenome.nih.gov/) to examine the TCTP protein and mRNA distribution. To explore the expression pattern of TCTP in liver cancer, we downloaded the expression profile of 50 liver cancer samples and 50 matched adjacent normal samples from the TCGA database (https://www.cancer.gov). Box plot presentation of TCTP mRNA expression level between cancer and normal samples showed the lower expression pattern in cancer samples (*P* value = 0.0025, Fig. 2A). Expression correlation analysis also revealed the similar low expression tendency of TCTP mRNA in HCC samples. To confirm the TCGA results, we then examined the TCTP protein levels (Fig. 2B) and mRNA levels (Fig. 2C) of the samples from HCC patients in our center by western blot and quantitative polymerase chain reaction (qPCR). Comparing with the *para*-tissues, the TCTP mRNA levels in the HCC tissues were found to be much lower, whereas the protein levels were higher in tumors. The results were consistent with the analysis based on the TCGA database. The contrary between TCTP and internal control alpha-fetoprotein mRNA levels verified lower mRNA levels of TCTP in the HCC tumors than *para*-tissues (Fig. 2D). By quantification of TCTP mRNA and protein levels, HCC tumors and *para*-tissues formed two significant different clusters, further showing that TCTP protein and mRNA were accumulated to high levels in HCC tumors and *para*-tissues, respectively (Fig. 2E). To mimic the in vivo environment of HCC and adjacent non-tumor tissues, HCC cell line Huh7 and normal hepatic cell line HL-7702 was chosen and the TCTP protein (Fig. 2F) and mRNA expression (Fig. 2G) levels in these two cell lines were measured. The results obtained in cells also demonstrated the existence of contradictive distribution of TCTP mRNA and protein, which was in accordance with the findings in HCC tissues.

### TCTP knockdown effectively reduces TCTP mRNA levels rather than protein levels in vitro

**Fig. 3:**
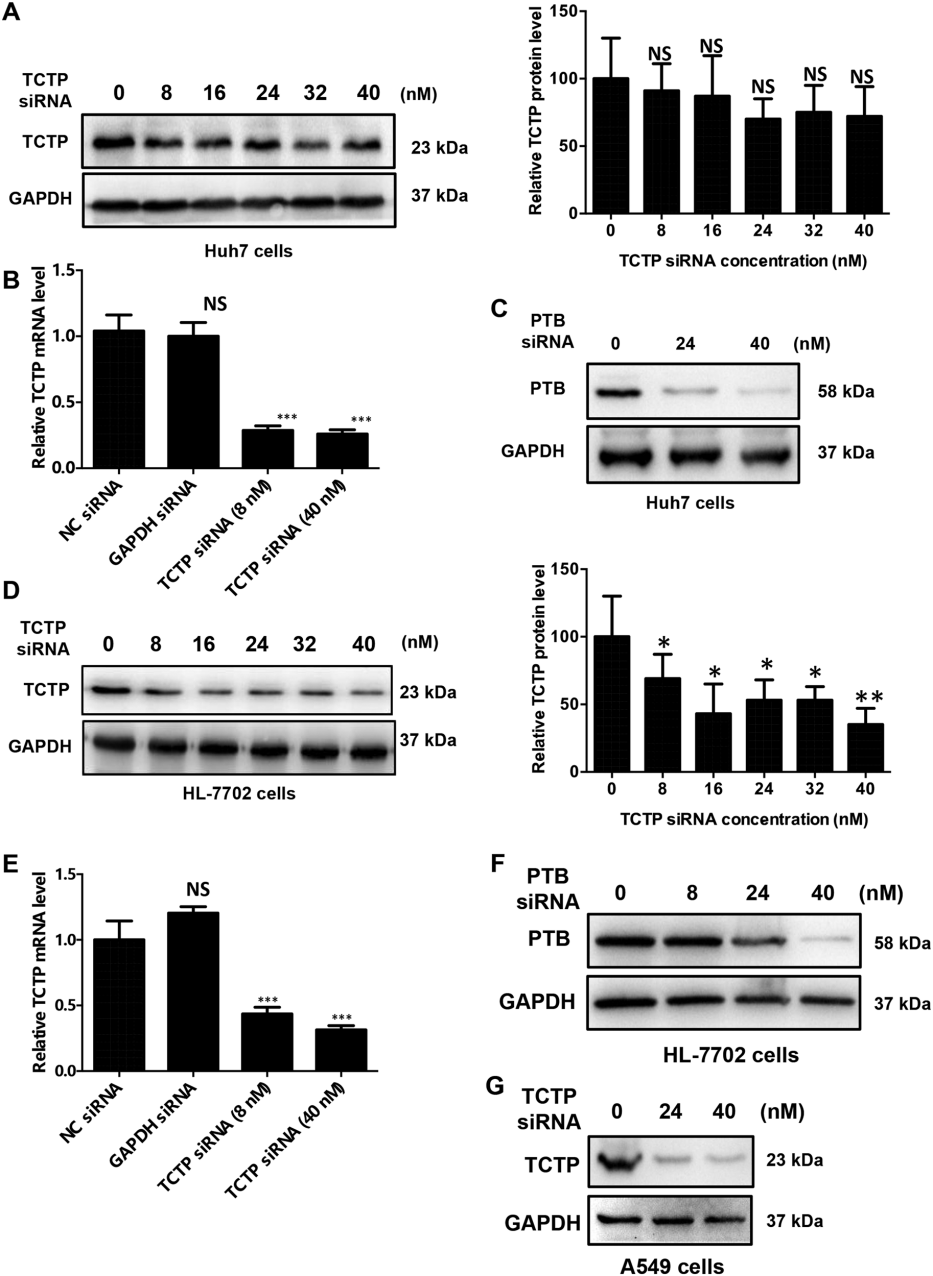
TCTP mRNA knockdown cannot effectively reduce TCTP protein levels in HCC cells. **A**, **B** TCTP protein and mRNA expression levels in Huh7 cells after Different concentrations of siRNAs against TCTP were transfected with 48 h. GAPDH was used as an internal control. **C** PTB protein levels in Huh7 cells after different concentrations of siRNAs against TCTP were transfected with 48 h. **D**, **E** Different concentrations of siRNAs against TCTP were transfected into HL-7702 cells. 48 h later, western blot (**D**) and qPCR (**E**) were used to detect the TCTP protein and mRNA levels, respectively. **F** PTB protein levels after treatment with different doses of siRNA against PTB in HL-7702 cells. **G** TCTP protein levels after treatment with different doses of siRNA against TCTP in A549 cells. The experiments were performed in triplicate and GAPDH was used as an internal control for all experiments. The data are expressed as means ± sd. Statistical analyses using Student’s *t* test for independent groups were performed using GraphPad Prism5. *P < 0.05, **P < 0.01, ***P < 0.001.

The contrary expression of TCTP mRNA and protein implies that low expression levels of TCTP mRNA could encode higher TCTP protein than normal or even high levels of TCTP mRNA in HCC. Therefore, the inhibition of TCTP may not certainly lead to its protein repression. To verify our hypothesis, we checked the TCTP protein level change caused by gradient knockdown (KD) of TCTP with different concentrations of siRNA against TCTP in both Huh7 HCC cells and HL-7702 normal hepatic cells. As shown in Fig. 3A, the TCTP protein level was only slightly decreased in Huh7 cells, although the TCTP mRNA was reduced efficiently. The mRNA KD effect reached a peak at low siRNA concentration (8 nM) (Fig. 3B) but the protein level kept stable even that the concentration of siRNA reached to 40 nM. In contrast, the protein levels of the control gene PTB were significantly decreased after treatment with different doses of siRNAs targeting PTB (Fig. 3C). As for HL-7702 cells, the similar TCTP mRNA KD trend was also observed following transfection with siRNAs (Fig. 3E), but the reduction of TCTP protein was still weak (Fig. 3D) when comparing with the effect of siRNA targeting PTB at various concentrations (Fig. 3F). However, the same siRNAs could target and KD TCTP protein efficiently in A549 non-small lung cancer cell lines (Fig. 3G), indicating that the inefficient KD of TCTP protein seems to be gene and cell specific. Thus, these results demonstrated that TCTP KD effectively reduced TCTP mRNA levels but not protein levels in HCC cells. Compared with the normal liver cell line (HL-7702), the protein level in the cancer cell line was only decreased at a very high level. This implies the existence of a cancer-specific mechanism. It means that the mRNA translation efficiency could be enhanced when the TCTP mRNA level decreased, thereby preventing HCC from losing or even obtaining more TCTP protein. This buffer system also explains the dramatic reduction of TCTP protein level in cancer cell line at 40 nm dose of siRNA: with the higher siRNA concentration, the mRNA will be reduced sharply, and therein cannot express enough TCTP protein even in the cancer cell.

### Low levels of TCTP mRNA can induce higher protein expression in vivo

Since TCTP can be released in a paracrine manner and contribute to distant tumor invasion and metastasis in vivo^23^, it is possible that the function of TCTP in transplanted cells was compensated by secreted TCTP from distant cells. To exclude the background interference, we generated TCTP KD transgenic mice. Gene targeting technology was applied to insert a neo-GFP reporter cassette into the third intron of the TCTP gene, and a stop codon was located in the 3′ part of insertion and interrupt the TCTP translation (Supplementary Fig. S2A). The KO construct targeted the TCTP locus in mouse embryonic stem cells, which was confirmed by PCR (Supplementary Fig. S2B), western blot (Supplementary Fig. S2C), and Southern blotting (Supplementary Fig. S2D). Germline-transmitted mice could be conveniently genotyped by PCR using a set of primers (Supplementary Fig. S2E). Homozygous TCTP KO (TCTP−/−) resulted in embryonic fetal, only heterozygous (TCTP+/−) ones can survive^24^. Although a rough 1:2 ratio of TCTP protein was detected in all tissues, the heterozygous mice (TCTP+/−) were phenotypically normal compared to WT C57BL mice with different genetic backgrounds (Supplementary Fig. S2F).

**Fig. 4:**
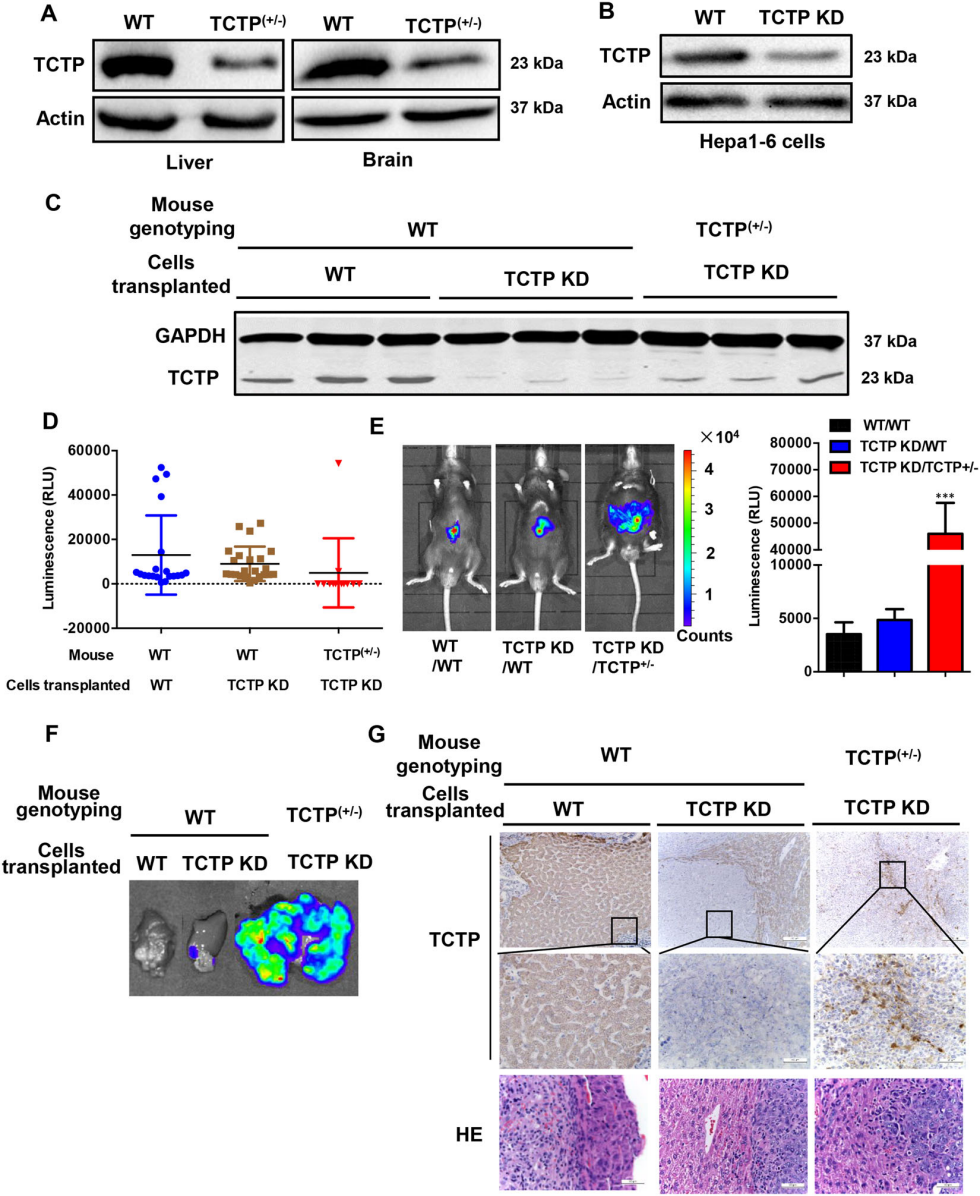
Low levels of TCTP mRNA may induce higher protein expression in vivo. **A** TCTP protein levels in liver and brain tissues of wildtype and heterozygous (TCTP+/−) mouse. **B** TCTP protein levels in wildtype and TCTP constitutive knockdown Hepa1-6 cells (the relative quantification of protein level was shown in Fig. S4A. **C** Wildtype and TCTP KD Hepa1-6 cells were transplanted in the livers of wild-type C57BL mice or TCTP+/− transgenic mice to generate three orthotopic tumor models, WT/WT, TCTP KD/WT, and TCTP KD/TCTP+/−. Western blot to test the TCTP protein level in transplanted in situ tumors from different tumor models. **D** Scatter plots of reads of bioluminescence intensities from the tumors in different genetic backgrounds mice. **E** Representative bioluminescence image of the different genetic backgrounds of mice with in situ tumors. Data presented as mean ± SD for the three independent experiments. ***p < 0.001 vs. control. **F** Representative images of livers loading the transplanted tumors excised from the three genetic background mice. **G** IHC and HE staining of the transplanted tumors and its *para* liver tissue. The edge of the exogenous cells and tissue cells can be observed in HE staining slides.

Western blot analysis demonstrated that TCTP protein was successfully KD in liver and brain tissues of heterozygous (TCTP+/−) mouse (Fig. 4A). As a control, WT and TCTP constitutively KD (TCTP KD) Hepa1–6 stable cell line labeled with luciferase were established (Fig. 4B) and transplanted in the livers of WT C57BL mice or TCTP+/− transgenic mice to generate three orthotopic tumor models, including WT cells transplanted in WT mice (WT/WT), TCTP KD cells in WT mice (TCTP KD/WT) and TCTP KD cells in TCTP+/− mice (TCTP KD/TCTP+/−) (40 mice for each tumor model). Comparing with WT/WT tumors, the TCTP protein expression levels in tumors formed by TCTP KD Hepa1–6 cells were repressed in both WT mice and the heterozygous TCTP+/− mice (Fig. 4C). Interestingly, in the low-density background, the TCTP protein levels in TCTP KD/TCTP+/− mice were a little higher than that in the TCTP KD/WT mice. The whole-body bioluminescence imaging was taken to monitor the growth of the in situ tumors two weeks after transplantation. The imaging results showed that the low expression of TCTP protein in TCTP KD/WT and TCTP KD/ TCTP+/− mice dramatically limited the transplanted tumor growth (Fig. 4D). Specifically, approximately 85% of heterozygous TCTP mice (TCTP KD/TCTP+/−) failed to develop tumors comparing with the 90% tumor formation ratio in NC/WT mice and 73% ratio in TCTP KD/WT, indicating that low TCTP protein level can repress the tumor formation. Notably, transplanting TCTP KD cells to the heterozygous TCTP mice (TCTP KD/TCTP+/−) resulted in the formation of abnormal larger tumors with a small probability (about 15%, 6 in 40 mice) comparing with the other two groups of wildtype mice (Fig. 4E, F). After measuring TCTP protein levels of the tumors from different genotype mice (Fig. 4G), we found that although the constitutive KD of TCTP repressed almost 80% of TCTP protein expression levels in tumors of wildtype mice (TCTP KD/WT), partial restoration of TCTP expression level was observed in the tumors of heterozygous TCTP mice (TCTP KD/TCTP+/−). These results indicated that low mRNA levels of TCTP may trigger a higher expression of TCTP protein.

### Low levels of TCTP mRNA can enhance its translational efficiency

**Fig. 5:**
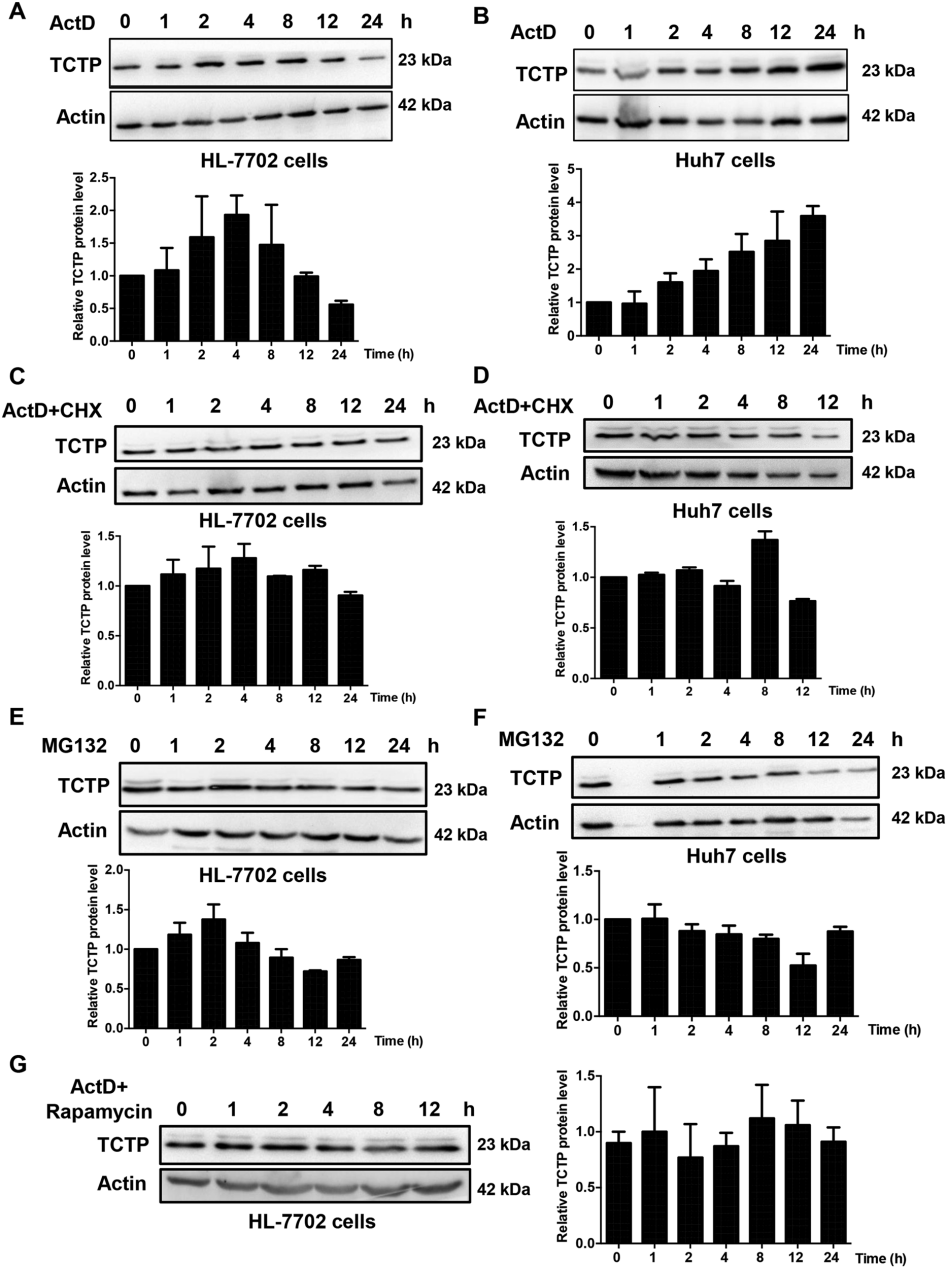
Low TCTP mRNA can enhance its translational efficiency. **A**, **B** The **A** HL-7702 or **B** Huh7 cells were treated with ActD (10 μg/ml) for different times, and western blot was used to detect the TCTP protein level in both cells. **C**, **D** The **C** HL-7702 or **D** Huh7 cells were treated with ActD (10 μg/ml) and CHX (10 μg/ml) for different times and western blot was used to detect the TCTP protein level in both cells. **E**, **F** The **E** HL-7702, and **F** Huh7 were treated with MG132 (1 μM) for a different time. Western blot was taken to detect the TCTP protein level in both cells. **G** The HL-7702 cells were pretreated with rapamycin (10 μM) for 1 h and then co-incubated with ActD (10 μg/ml) for a different time. Western blot was then taken to detect the TCTP protein level in cells. The experiments were performed in triplicate and actin was used as an internal control for all experiments.

To explore whether the low mRNA level of TCTP induces the increase of translational efficiency, TCTP protein levels were measured in different cell lines after blocking its mRNA synthesis. Both HL-7702 (Fig. 5A) and Huh7 (Fig. 5B) cells were treated with actinomycin D (ActD), which inhibits polymerase II-dependent transcription, for different time periods. Without new mRNA synthesis, the TCTP protein levels in both cell lines were increased. Interestingly, the protein level in HL-7702 cells reached a peak after 4 h of ActD treatment, then began to decrease afterward. Twenty-four-hour later, only half of TCTP protein in HL-7702 cells was left compared with that at 0 h time point. In contrast, the TCTP protein in Huh7 cells was consistently increased with time. These results may explain why TCTP protein was accumulated with a low mRNA level in HCC (the mRNA level was shown in Fig. S4D).

To further prove the TCTP protein accumulation was caused by translational efficiency increase, the cells were treated with ActD, as well as cycloheximide, which blocks protein synthesis. As shown in Fig. 5C, D, the accumulation of protein induced by ActD treatment disappeared in both cell lines. Whereas, treatment with proteasome inhibitor MG132, which is supposed to repress the proteasomal degradation pathway, did not lead to TCTP protein accumulation (Fig. 5E, F). The same trends of protein metabolism can be observed in both cell lines, indicating that the stability of TCTP protein is similar in HCC tumor condition and normal liver condition. Given that previous studies have reported that the translational regulation of TCTP mRNA can be regulated through the PI3-K/Akt/mTORC1 signaling pathway^22^, we tried to figure out whether mTORC1 pathway was involved in the ActD-induced TCTP protein accumulation. The HL-7702 cells were pretreated with rapamycin for 1 h and then co-incubated with ActD for a different time. As showed by western blot analysis, the TCTP protein level had no obvious change over time (Fig. 5G), indicating that the protein accumulation induced by ActD treatment was involved in PI3-K/Akt/mTORC1 signaling pathway.

### Exogenous TCTP mRNA keeps a stable level of TCTP protein

**Fig. 6:**
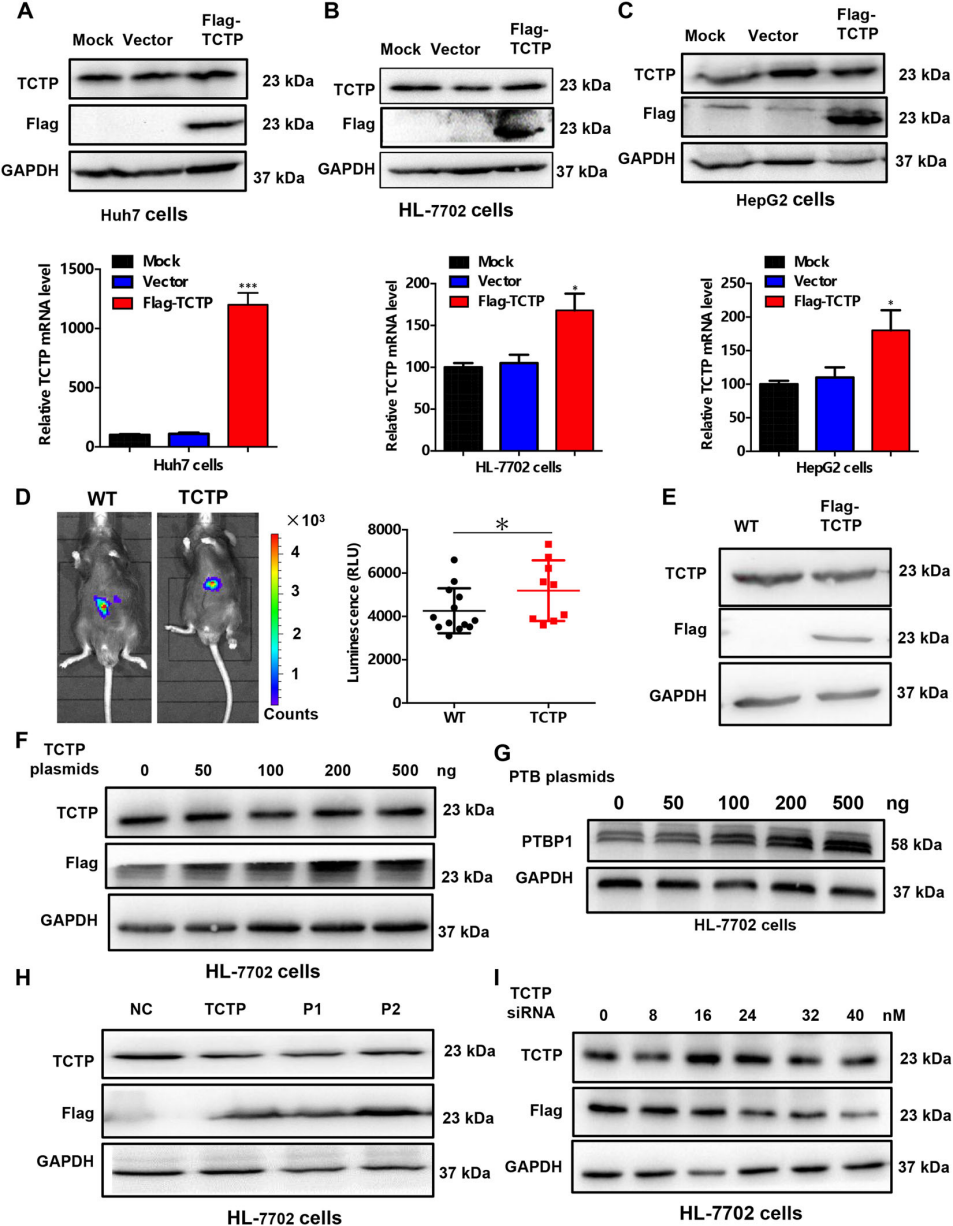
Overexpressed TCTP mRNA keeps its protein expression at a stable level. **A**–**C** Flag-tagged TCTP overexpressed plasmids (Flag-TCTP) and empty vector control (Vector) plasmids were transfected in **A** Huh7 cells, **B** HL-7702 cells, and **C** HepG2 cells. Forty-eight hour later, western blot and qPCR was taken to test the TCTP protein (upper) and mRNA (bottom) levels, respectively. **D** Wildtype (WT) and TCTP constitutive overexpressed Hepa1–6 cells with luciferase were transplanted in the livers of C57BL mice to establish orthotopic tumor models. The in vivo bioluminescence imaging was taken 2 weeks after transplantation. And the luminescence intensities were quantified. **E** TCTP and Flag protein expression levels in the tumor samples from the two mice model. **F** Different concentrations of TCTP overexpressed plasmids (pTCTP) were transfected in HL-7702 cells. Forty-eight hours later, western blot was used to detect the TCTP and Flag protein expression. **G** Different concentrations of PTB overexpressed plasmids (pPTB) were transfected in HL-7702 cells. Forty-eight hour later, western blot was used to detect the PTBP1 expression. **H** TCTP and Flag expression levels in the TCTP constitutively overexpressed HL-7702 cells (TCTP), as well as the cells transfected with TCTP overexpressed plasmids under the control of different promoters (P1 and P2). **I** Gradient concentrations of siRNA against TCTP was transfected in the TCTP constitutive overexpressed HL-7702 cells. Forty-eight hours later, a western blot was taken to check the expression level of TCTP and Flag. GAPDH was used as an internal control for all experiments and the experiments were performed in triplicate.

Since low mRNA level of TCTP can regulate its own protein expression, we intend to investigate whether high mRNA levels of TCTP could also increase the corresponding protein levels. Therefore, TCTP-overexpressed plasmids were introduced into different HCC cell lines and the expression levels of TCTP mRNA and protein were investigated. Surprisingly, we found that the mRNA levels were significantly increased in the HCC cells with transfection of exogenous TCTP plasmids. The TCTP protein level, on the contrary, did not change with the recombination TCTP expression and kept a rather stable level (Fig. 6A–C, the relative quantification of protein level was shown in Fig. S4B). The expression of Flag tag proved that the plasmids were successfully transfected and TCTP mRNA was upregulated in all cell lines. To verify the function of the overexpressed TCTP mRNA in vivo, the WT and constitutively overexpressed Hepa1–6 cells with luciferase were transplanted into the livers of C57BL mice to establish orthotopic tumor models. The in vivo bioluminescence imaging results showed that no obvious difference of signal intensity was observed between these two mice models (Fig. 6D), indicating that overexpression of TCTP mRNA did not promote tumor growth and proliferation. Western blot analysis of the tumor samples demonstrated that TCTP-overexpressed cells were successfully transplanted while the corresponding protein was not increased (Fig. 6E). IHC images confirmed that the ectopic expression of TCTP mRNA did not upregulate the TCTP protein level, let alone enhance the tumor proliferation (Supplementary Fig. S3).

To further explore the effect of exogenous TCTP on protein expression, the gradient concentrations of TCTP-overexpressed plasmids were transfected into HL-7702 cells. With the increasing amounts of transfected plasmids, the expression level of recombinant protein with Flag-tag was increased gradually. However, the total TCTP protein level was almost unchanged in the whole process (Fig. 6F). In contrast, the PTBP1 protein expression was increased according to the gradient increase dose of PTBP1 overexpressed plasmids in HL-7702 cells (Fig. 6G). To exclude the effect of plasmid transfection or promoters on the TCTP protein expression, the TCTP protein levels were investigated in the TCTP constitutively overexpressed HL-7702 cells, as well as the cells transfected with TCTP-overexpressed plasmids under the control of different promoters (P1 or P2). The TCTP protein level had no obvious change although the Flag tagged-recombinant protein was upregulated (Fig. 6H). We then knocked down TCTP by siRNAs in TCTP constitutively overexpressed HL-7702 cells and checked the protein change. As shown in Fig. 6I, Flag tagged-recombinant protein levels was reduced in a dose-dependent manner but TCTP protein change was very mild. Collectively, these results suggested that exogenous TCTP may inhibit the endogenous mRNA expression and keep a stable level of TCTP protein under the TCTP mRNA overexpressed condition.

### The coding sequence of TCTP mRNA functions as a sponge to repress its own protein expression

**Fig. 7:**
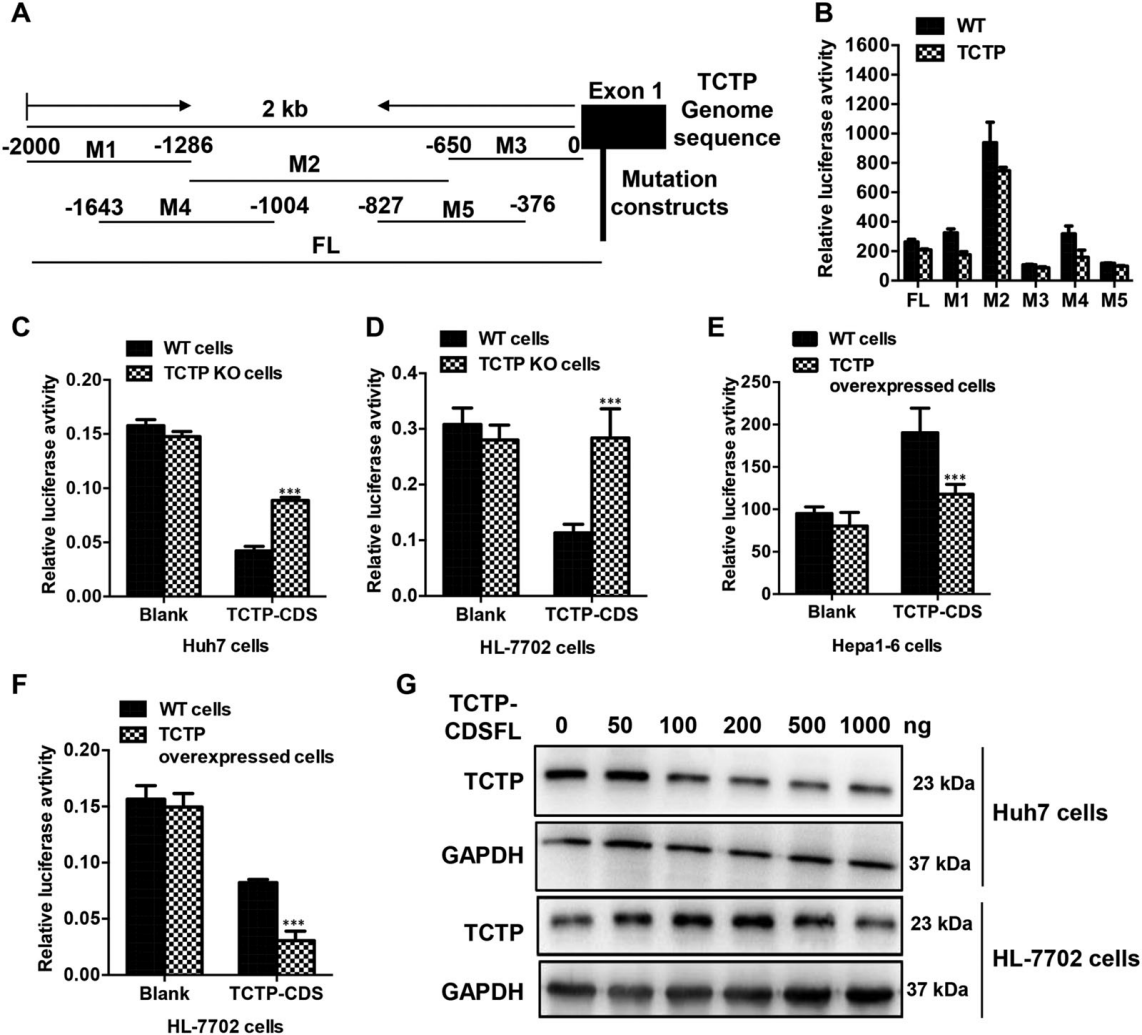
Coding sequence of TCTP mRNA represses its own translation. **A** The 2 kb DNA sequence of the TCTP promoter. Different truncated sequences (M1–M5) are labeled for the mutation construct. The full length (FL) and five mutations (M1–M5) were inserted to the 5′UTR of a pGL3-basic vector to construct a series of luciferase reporters. **B** The wildtype and TCTP constitutively overexpressed Hepa1-6 cells were transfected with the FL and mutated reporters (M1–M5), as well as a control luciferase plasmid pRL-TK. Forty-eight hours later, dual-luciferase assay was employed to detect the relative luciferase activities. **C**–**F** TCTP-CDS luciferase plasmids and blank control plasmids were transfected in **C** TCTP KO and wild-type Huh7 cells, **D** TCTP KO and wild-type HL-7702 cells, **E** TCTP overexpressed and wildtype Hepa1–6 cells, **F** TCTP overexpressed and wild-type HL-7702 cells. Forty-eight hours later, dual-luciferase assay was employed to detect the relative luciferase activities. **G** Different concentrations of TCTP-CDSFL plasmids were transfected in Huh7 cells or HL-7702 cells. Forty-eight hours later, western blot was used to detect the TCTP protein expression. GAPDH was used as an internal control for all experiments and the experiments were performed in triplicate. Data presented as mean ± SD for the three independent experiments. ***p < 0.001 vs. control.

TCTP protein has been reported as a transcription regulator and can regulate the mRNA level by regulating the transcription^25^. To exclude the possibility that the repression of TCTP protein was caused by negative feedback on TCTP mRNA transcription, the full length (FL) sequence of TCTP promoter (2000 bp upstream from the first nucleotide of exon 1) and another five fragments (M1–M5) containing different sizes of the TCTP promoter were individually cloned upstream of a promoterless luciferase reporter vector (Fig. 7A). The FL reporter and various mutated reporters (M1–M5) were transfected into the TCTP constitutively overexpressed Hepa1–6 cells and WT cells. As revealed by the luciferase assay, neither the FL reporter nor the truncated mutations (M1–M5) showed obvious luciferase activities change between the TCTP-overexpressed cells and WT cells (Fig. 7B), indicating that the TCTP protein repression may not be subjected to regulation at the transcription level.

To investigate the coding-independent function of TCTP mRNA sequence, the sequence of exon 4 and exon 5 of TCTP was inserted into the 3′ UTR of a luciferase reporter to obtain the TCTP-CDS construct, which abandoned the coding function of TCTP. These two exons were constitutively spliced in all isoforms. The TCTP-CDS and blank reporters were transfected into different cell lines that TCTP was constitutively KO or overexpressed. The TCTP-CDS construct induced a remarkable increase of luciferase activities in TCTP KO cell lines compared with that in WT cells (Fig. 7C, D). On the contrary, in the TCTP-CDS construct transfected cells, a significant decrease of luciferase signal can be observed in the TCTP-overexpressed Hepa1–6 and HL-7702 cells but not in respective wildtype cells (Fig. 7E, F). These results suggested that the sequence of TCTP mRNA may regulate the TCTP protein expression in a coding-independent way.

We finally examined whether the existence of exogenous TCTP mRNA without translation function can repress the endogenous TCTP protein expression. FL of TCTP CDS sequence was inserted into the 3′ UTR of a luciferase reporter to generate TCTP-CDSFL construct. With gradient amounts of TCTP-CDSFL plasmids transfected into cells, continuous inhibition of endogenous TCTP protein expression was observed in Huh7 cells (Fig. 7G). As in HL-7702 cells, the effect of TCTP-CDSFL showed a different trend, upregulating the TCTP protein at the beginning then downregulating the protein expression at high concentration (1 μg/well). Together, the TCTP mRNA sequence may have a coding-independent regulatory function and play an important role in keeping TCTP protein stable.

## Discussion

High expression levels of TCTP have been observed in various tumors and shown to function diversely during tumorigenesis. As a known tumor reversal target, high density of TCTP protein is highly necessary for tumor development and metastasis. Compared with the clear function of TCTP protein, the distribution of TCTP mRNA and its role in carcinogenesis was rather confused. In some tissues, the accumulation of TCTP mRNA has been reported without an increase in its corresponding protein level^26^. The inhibition of TCTP mRNA was caused by the ribonucleoproteins (RNPs) is bound to. TCTP mRNA is stored in the cytoplasmic untranslated messenger RNP particles and can rapidly induce the TCTP synthesis rate^27^. Furthermore, translation inhibition was tightly restricted by its high structure. A 5′-TOP structure is located on the 5′-UTR of TCTP mRNA and the translation repression could be released through the mTORC1 pathway. Higher levels of TCTP mRNA are not accompanied by higher levels of protein, and reducing the TCTP mRNA level does not lead to a decrease in protein levels^28^. The complex relationship between TCTP mRNA and protein levels implies the TCTP protein expression is regulated by multiple mechanisms rather than only dependent on its translation function.

In all, we found a contradiction between the mRNA and protein expression of TCTP in HCC cells and tissues. Although the accumulation of TCTP mRNA was observed in non-tumor tissues, the protein levels were rather lower compared to the corresponding HCC tumor tissue. In contrast, the TCTP protein expression in tumor tissues was at a high level, but the corresponding mRNA level was extremely limited, much lower than that in the paired *para*-tissues. Our results suggest that TCTP mRNA can exert its function in a coding-independent manner, thereby balancing TCTP protein expression. A closed regulatory loop is formed between the TCTP mRNA and its protein to maintain the TCTP protein level stable. The TCTP mRNA/protein circuit can work as a buffer system to regulate the TCTP protein level according to TCTP mRNA concentration. When TCTP mRNA concentration is dropped to a very low level, higher translational efficiency of the mRNA will be stimulated and more TCTP protein will be synthesized both in vivo and in vitro. On the other hand, when the mRNA concentration is raised to a certain extent, rather than the accumulation of TCTP protein, both the increased TCTP mRNA and the protein itself will inhibit the translation and sequentially decrease the protein levels. Our findings also suggest that in addition to non-coding RNA, mRNA can also perform regulatory functions in a coding-independent manner. The strong relationship between the higher concentration of TCTP mRNA and the translational inhibition of TCTP protein indicates that the sponge model suitable for noncoding RNA^29^ and 3′ UTR^30^ is also suitable for mRNA. More importantly, the large-scale inconsistency between protein abundance and its encoded mRNA in humans^31,32^ implies that this coding-independent regulatory way is more likely to be a general mechanism rather than a specific way of a certain gene.

A buffer system formed between TCTP protein and mRNA protects the cells from accumulation or loss of TCTP protein to an extreme extent. This gives a hint to understand how the mammals maintain hemostasis in protein synthesis in vivo. In vitro protein expression efficiencies vary widely according to the different systems and different genes. It is impractical to increase the protein density without limitation by introducing exogenous coding sequences. It is not clear what mechanism is used to determine whether a certain protein is sufficient enough and when to stop its translation. In our study, we found that excess exogenous TCTP mRNA accumulates in cells rather than degrades. Moreover, these ineffective mRNAs (mRNAs with low translational efficiency) retain their expression potential. As long as the TCTP mRNA concentration is lowered, the translation potential is activated. The same phenomenon can be observed in vivo. This indicates that concentration-dependent inhibition is an intrinsic mechanism for maintaining TCTP protein homeostasis in vivo.

Our results indicate that TCTP mRNA exerts other potential functions more than just encoding its protein. A buffer system is formed between TCTP mRNA and protein to keep the TCTP protein level in a relatively stable status. The system allows the TCTP protein to resist certain external signal intensities, not easily increasing or decreasing TCTP protein levels. The elaborate system to keep the stability of TCTP abundance implies the necessity of the precise level of TCTP protein in HCC. Disrupting the balance of the buffering system will lead to the abnormal level of TCTP protein and susceptible to HCC genesis. This study provides a new perspective for understanding the regulation of TCTP expression. Our finding also increases the difficulty of therapeutics for targeting TCTP, since lower TCTP mRNA in HCC may have higher translational efficiency. Targeting TCTP mRNA with siRNA is not certain to reduce TCTP protein levels, and the attempt to reduce TCTP protein may even trigger higher TCTP protein levels. This is consistent with clinical and research evidence, which suggests that both high and low expression of TCTP may result in abnormal physical condition^33^.

In conclusion, our results proved that TCTP mRNA can regulate its protein expression in a coding-independent manner. TCTP mRNA levels act as regulatory factors and control the translational efficiency of mRNA itself in a negative feedback manner. The TCTP mRNA and protein work as a buffer system to keep the homeostasis of the TCTP protein level in vivo. Disrupting the balance of this system will lead to abnormal expression of TCTP, which in turn promotes the progression of HCC.

## Supplementary information

Supplementary Fig S1

Supplementary Fig S2

Supplementary Fig S3

Supplementary Fig S4

Supporting information
