# Supplementary figures and images for: The mRNA of TCTP functions as a sponge to maintain homeostasis of TCTP protein levels in hepatocellular carcinoma
Source: Cell Death Dis. 2020 Nov 12;11(11):974. doi: 10.1038/s41419-020-03149-7 (PMC7665032; doi:10.1038/s41419-020-03149-7)

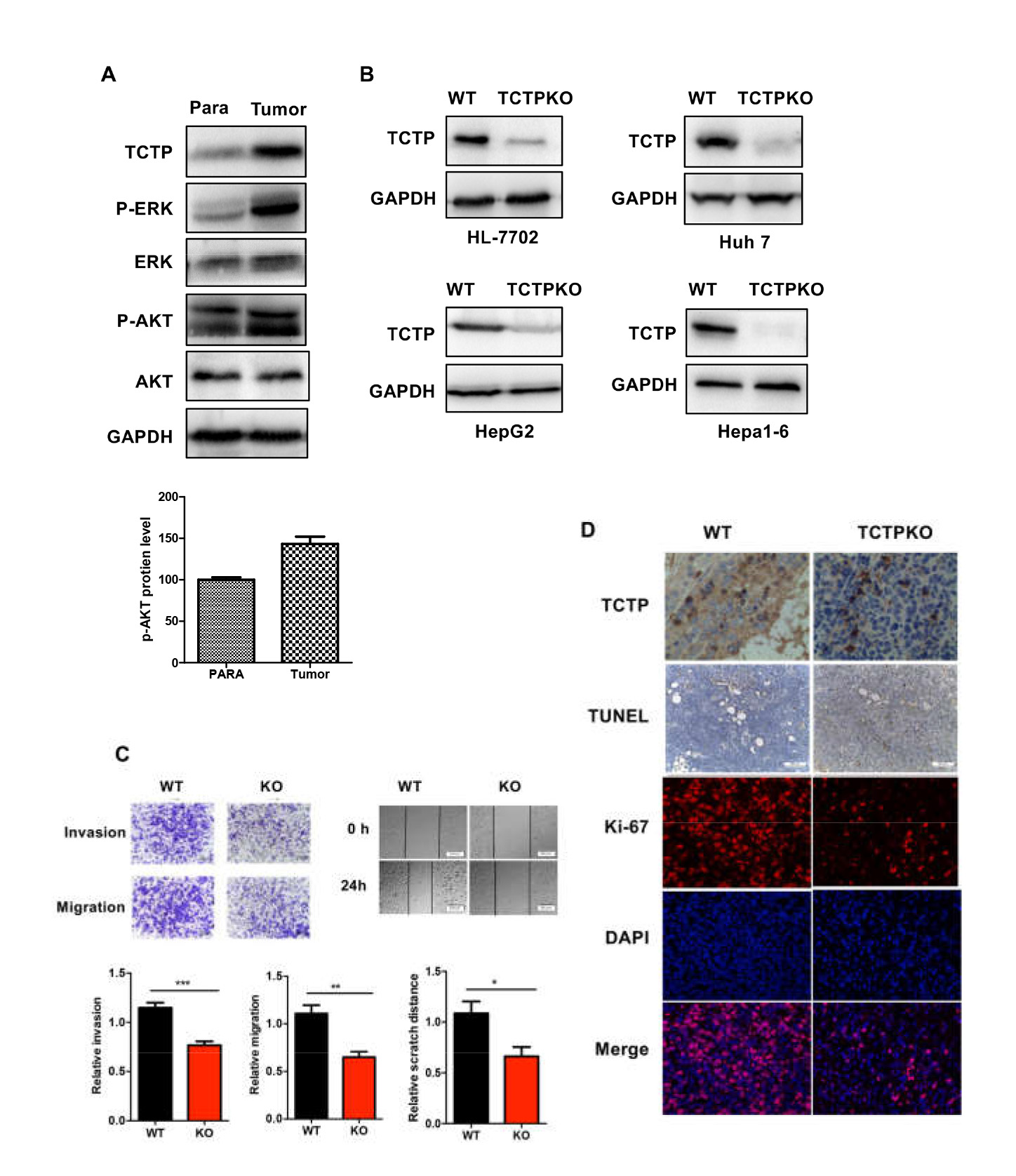

Supplement: Supplementary file 1 — Supplementary Fig S1 [file 41419_2020_3149_MOESM1_ESM.tif]

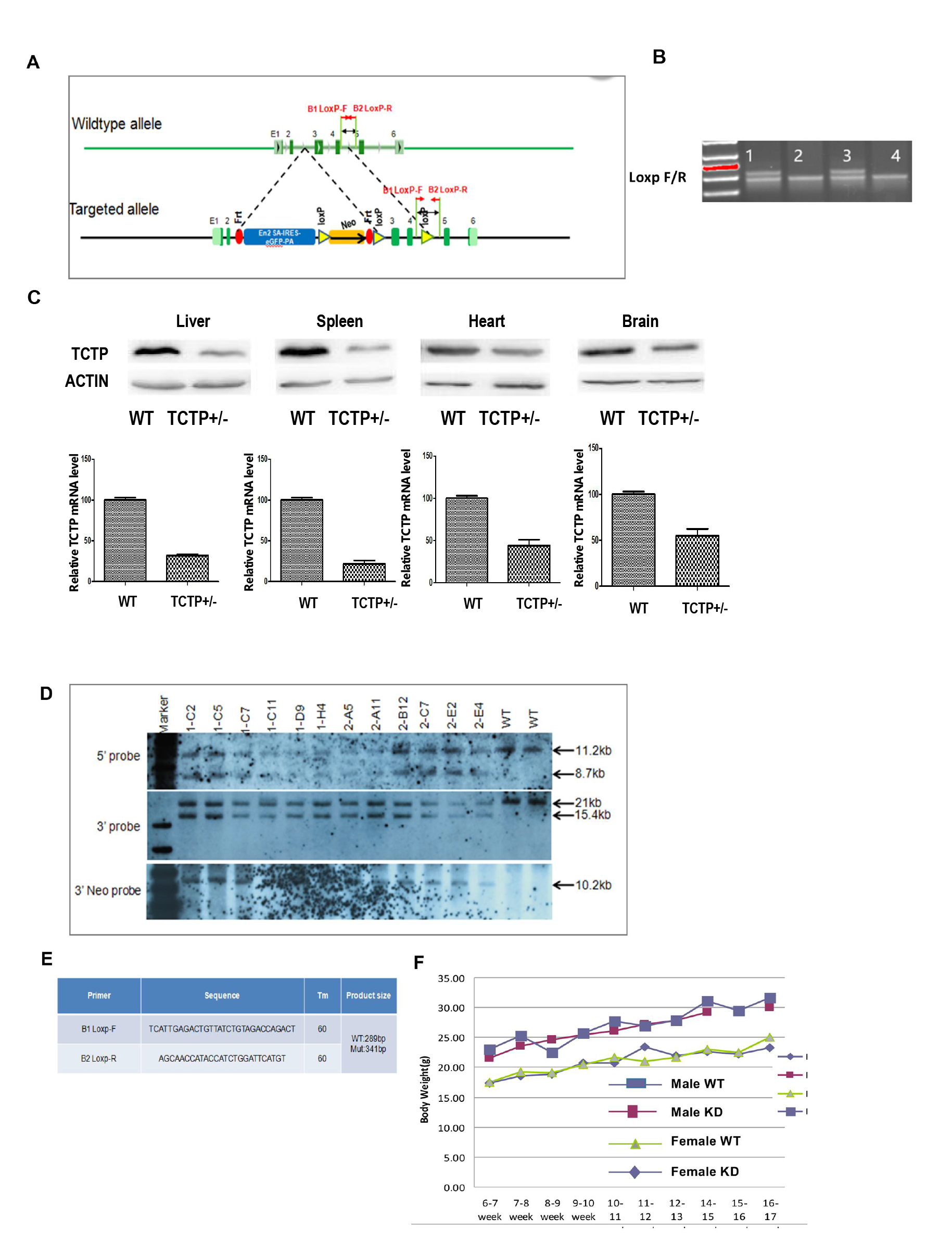

Supplement: Supplementary file 2 — Supplementary Fig S2 [file 41419_2020_3149_MOESM2_ESM.tif]

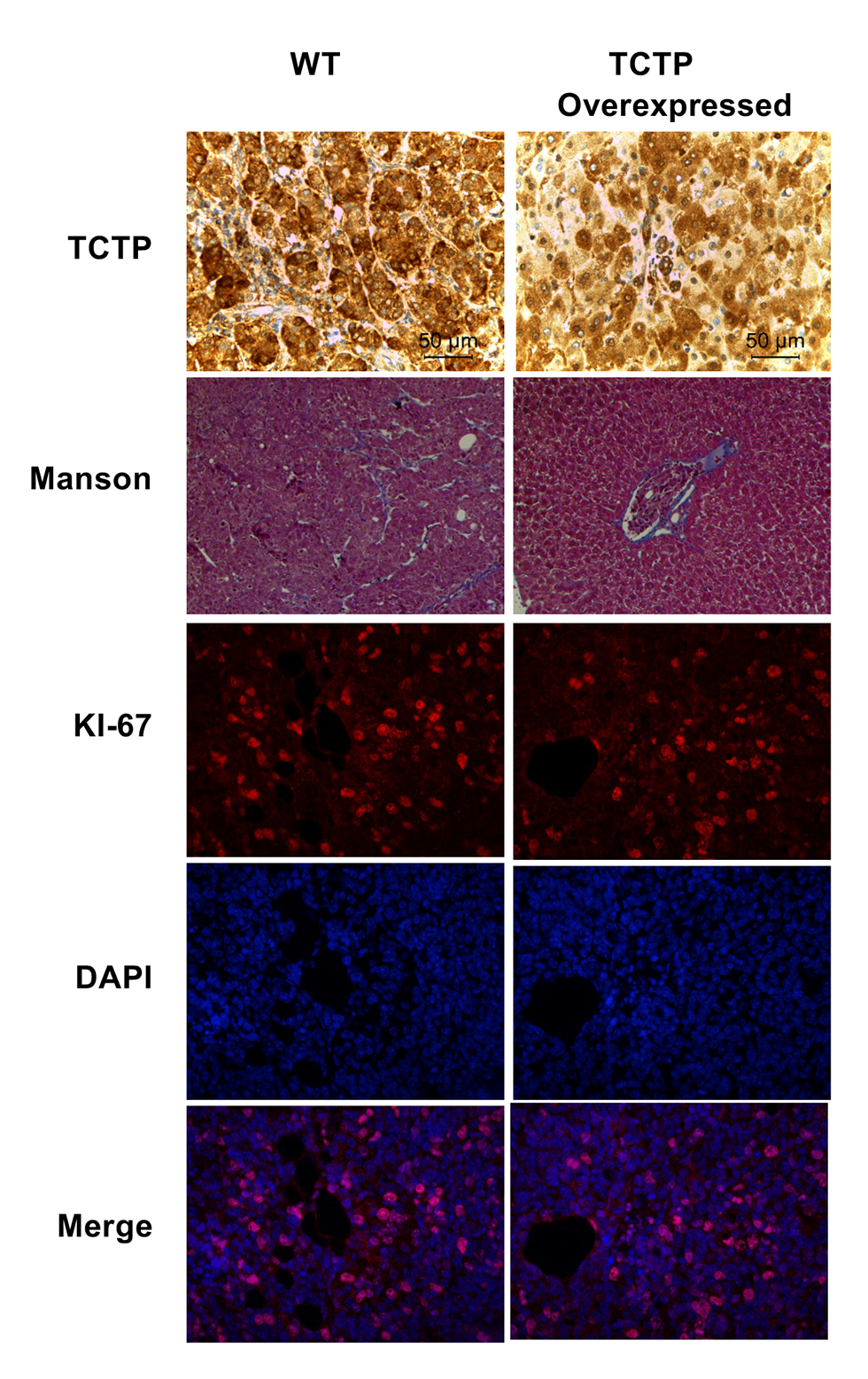

Supplement: Supplementary file 3 — Supplementary Fig S3 [file 41419_2020_3149_MOESM3_ESM.tif]

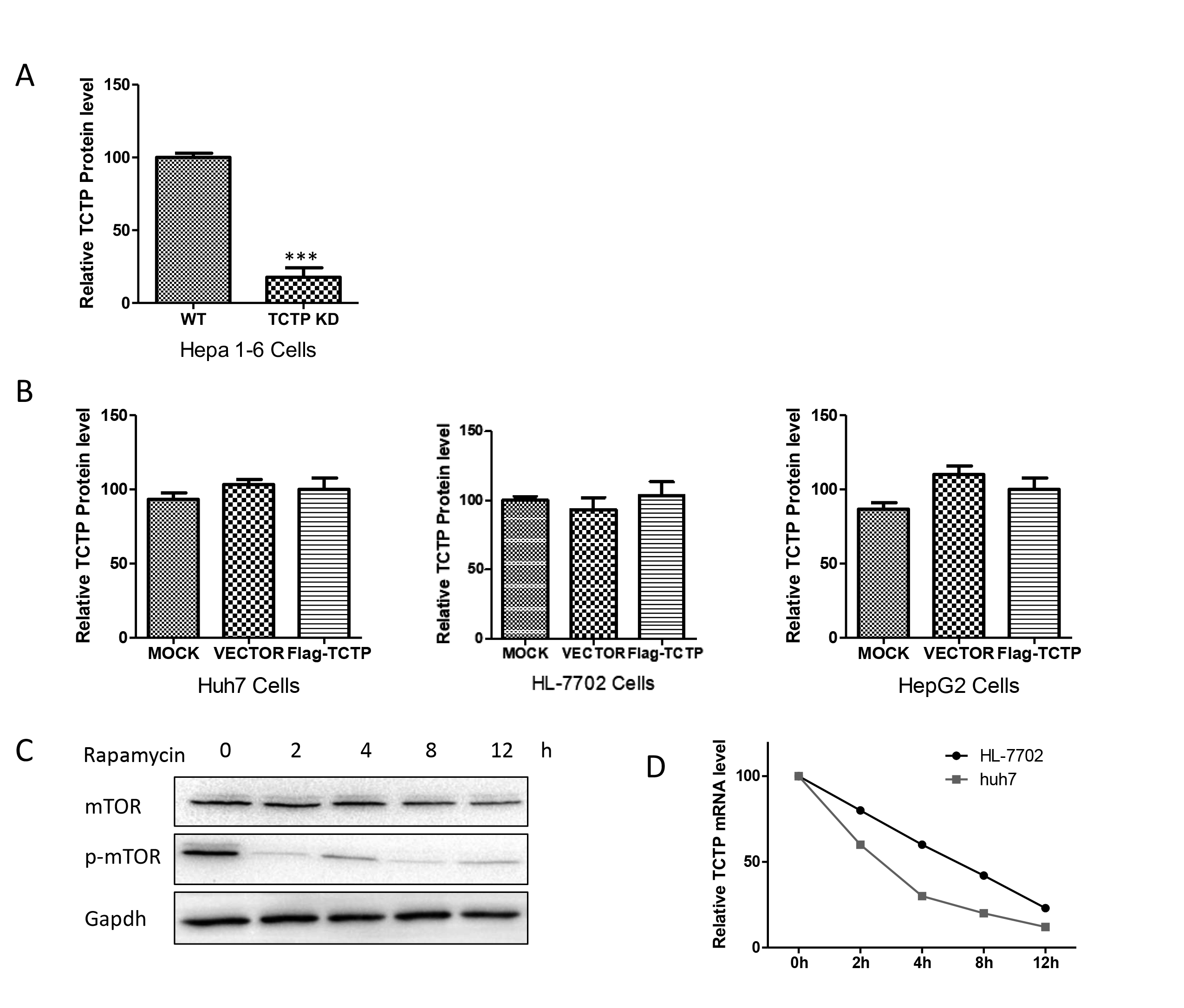

Supplement: Supplementary file 4 — Supplementary Fig S4 [file 41419_2020_3149_MOESM4_ESM.tif]
