# Supplementary material for: The mRNA of TCTP functions as a sponge to maintain homeostasis of TCTP protein levels in hepatocellular carcinoma
Source: Cell Death Dis. 2020 Nov 12;11(11):974. doi: 10.1038/s41419-020-03149-7 (PMC7665032; doi:10.1038/s41419-020-03149-7)
Supplement: Supplementary file 5 — Supporting information [file 41419_2020_3149_MOESM5_ESM.docx]

**Supporting information**

**Supplementary Figure Legends**

**Figure S1.** (A) Protein expression of TCTP, ERK, AKT, phosphorylated ERK (P-ERK), phosphorylated AKT (P-AKT) in paired HCC tumors and para-tissues. GAPDH was used as an internal loading control. (B) Protein expression of TCTP in HL-7702, Huh7, HepG2 and HL-7702 four different hepatic cell lines with TCTP knockout using CRISPR/Cas9 lentivirus system. (C) The migration and the invasion abilities of WT and TCTP KO Hepa1-6 cells were investigated by Transwell invasion assay (top), Transwell migration assay (middle) and wound healing assay (bottom). (D) Representative IHC images of TCTP and Ki-67 expression in tumor samples from Hepa1-6 WT and TCTP KO xenograft tumor model. DAPI was used to stain nuclei.

**Figure S2.** (A) Schematic of knocking down the TCTP gene based upon gene targeting technology. (B) Genotyping of four different samples from TCTP +/- transgenic mice using PCR. (C) TCTP protein (upper) and mRNA (lower) expression in different tissues including liver, spleen, heart and brain from TCTP +/- transgenic mice. (D) Genotyping of transgenic mice using Southern blotting with different probes. (E) Primers for genotyping by PCR. (F) Body weight of TCTP +/- transgenic mice with different genetic backgrounds over time.

**Figure S3.** IHC analysis of the TCTP and Ki-67 expression in the tumors from the wildtype and TCTP-overexpressed orthotopic mice models. DAPI was used to stain nuclei.

**Figure S4.** (A) Quantification of the TCTP protein relative level in wildtype and TCTP constitutive knockdown Hepa1-6 cells. (B) Quantification of relative TCTP protein level in Flag-tagged TCTP overexpressed plasmids (Flag-TCTP) and empty vector control (Vector) plasmids were transfected in Huh7 cells (left), HL-7702 cells (middle) and HepG2 cells (right) 48 h later. (C) The p-mTOR protein level in HL-7702 cells after rapamycin (10 μM) treatment for different time points. The experiments were performed in triplicate and actin was used as an internal control for all experiments. (D) The TCTP mRNA level in HL-7702 or Huh7 cells after treated with ActD (10 μg/ml) for different time.

**Supplementary Methods**

**Plasmid construction**

To overexpress TCTP protein *in vitro*, full length of TCTP coding sequence was synthesized *in vitro* (Shanghai Sangon Biotech, Inc, China) and inserted to pcDNA3.1 (Promega) and pEGFPN1 (Promega), respectively. These two overexpression vectors have different promoters. For analysis of the TCTP mRNA transcription, truncated TCTP coding sequence and full length coding sequence was synthesized in vitro (Shanghai Sangon Biotech, Inc, China) and inserted into the downstream of Renilla luciferase of psiCHECK2 vector (Promega). For the promoter activity analysis, truncated or full length of 2 kb of sequence upstream of the first nucleotide of exon 1 was amplified by PCR and inserted to pGL3-Basic vector, and obtained a series of plasmids containing the full length promoter (FL) or the truncated promoter sequence (M1-M5).

**qPCR and western blotting**

qPCR was performed with SYBR green master mix (Takara) according to the manufacturer’s instructions. Primers used to amplify TCTP were listed as follows. Human-TCTP-F: 5’-AGA CCA GAA AGA GTA AAA-3’; Human-TCTP-R: 5’-TCC ACT CCA AAT AAA TCA CAG-3’; Mouse-TCTP-F: 5’-GAG CTG CAG AGC AGA TTA AG-3’; Mouse-TCTP-R: 5’-CCA TCT TCA CGG TAG TCC AG-3’; AFP-F: 5’-CAG GAA GTC TGC TTT GCT GAA G-3’; AFP-R: 5’-TCA CAC CGA ATG AAA GAC TCG-3’; GAPDH-F: 5’- GCA CCG TCA AGG CTG AGA AC-3’; GAPDH-R: 5’-TGG TGA AGA CGC CAG TGG A-3’. Western blotting analysis was conducted with various specific primary and secondary antibodies. All the antibodies were purchased from Santa Cruz Biotechnology.

**Immunohistochemistry assay**

HCC tissue samples were dissected and fixed in 4% paraformaldehyde, then cut into 5 μm-thick paraffin-embedded sections. Sections were firstly deparaffinized with 100% xylene, followed by rehydration using gradient ethanol (100%, 95%, 70%, 30%, 0) for 5 min per time. After inactivation of endogenous peroxidase, sections were then incubated in 5% BSA for 30 min and probed with the respective primary antibodies (a final dilution of 1: 500 for TCTP, a final dilution of 1:1000 for Ki-67) at 4°C overnight. The HRP-conjugated secondary antibodies were then incubated for 4 h at room temperature. Hematoxylin was used for nuclear counterstaining.

**TCTP constitutive knockdown, knockout and overexpressing cell lines**

Lenti-virus system was used to constitutive knockdown, knockout and overexpress TCTP in different cell lines. Virus were designed to load siRNAs targeting TCTP, TCTP overexpressing plasmids and CRISPR/Cas9 system targeting genomic TCTP sequences, respectively. Two kinds of virus were employed, one labeled with GFP, the other with luciferase. The GFP or luciferase gene was located downstream of the functional sequence of TCTP. Cells infected with virus containing luciferase were used for living image.

**Generation of TCTP knockdown mice**

Specific restriction fragments containing TCTP genomic sequences were obtained by PCR amplification and cloned into the gene targeting vector (Supplementary Figure S2). Northern blot and western blot were used to characterize the knockout mice. The primer sequences used for mice genotyping were listed in supplementary figure S2E.

**Bioinformatics analysis**

TPT1 Gene expression data was downloaded from TCGA database (https://cancergenome.nih.gov/) to identify the transcription pattern of TPT1 in HCC. 50 pairs of patients’ samples (all the pairing samples in TCGA) were analyzed.

**Cell Viability**

To determine the cell viability, Cell Counting Kit-8 (CCK-8, Dojindo Laboratories, Japan) was performed. At the end of each experiment, 50 μl CCK-8 was added in each well for 3 h, and then the absorbance at 450 nm was measured by GloMax Discover and Explorer Detection Systems (Promega).
